# The Dual Impacts of Fathers’ Beliefs on Children’s Social Adjustment: Serial Mediation Models Connecting Father Involvement and the Father–Child Relationship

**DOI:** 10.3390/bs16050777

**Published:** 2026-05-14

**Authors:** Peishan Huang, Jiajun Mo, Liman Cai, Xiaojia Deng, Dengjun Liu

**Affiliations:** 1 School of Education (Shanwei), South China Normal University, Shanwei 516625, China; yc17122@umac.mo; 2 Guangzhou Institute of Educational Research, Guangzhou 510055, China; jiajun.mo@outlook.com; 3 School of Education, South China Normal University, Guangzhou 510631, China; liudengjun@m.scnu.edu.cn; 4 University International College, Macau University of Science and Technology, Taipa, Macao SAR 999078, China; xjdeng@must.edu.mo

**Keywords:** father belief about children, father involvement, father–child relationship, children social adjustment, China

## Abstract

Fathers are globally recognized as influential figures in children’s development, yet the specific mechanisms linking paternal beliefs to preschoolers’ social adjustment remain insufficiently explored. This study examined the sequential mediation effects of father involvement and the father–child relationship on the link between paternal progressive beliefs and children’s social adjustment (indexed by social competence and problem behaviors). A stratified random sample of 1862 Chinese mother–father dyads (3724 individual participants) was recruited. Structural equation modeling showed that the following: (1) Fathers’ progressive beliefs had a direct positive association with children’s social competence, and a small but significant direct positive link to children’s anger–aggression behaviors; (2) The associations between the fathers’ beliefs and children’s social adjustment were indirectly explained by a sequential mediation process: beliefs were associated with greater father involvement, which, in turn, connected to fostered closeness or increased father–child conflict, ultimately leading to more positive adjustments through closeness, or to more negative adjustments via conflict. This study also uncovered discrepancies between mothers’ and fathers’ perceptions of the fathering process. Notably, due to the lack of parallel measures of maternal constructs, these findings reflect paternal contributions within the family system rather than unique effects. These findings were discussed within the transitional context of culturally specific Chinese fathering. This study extends the traditional “parenting beliefs–practices–outcomes” framework to include the parent–child relationship, highlighting the importance of targeting fathers’ effective relationship-building practices in family programs.

## 1. Introduction

Preschoolers’ social adjustment, characterized by social competence (adaptive aspect), minimal anxiety–withdrawal, and anger–aggression (maladaptive aspect) (LaFreniere & Dumas, 1996), has been demonstrated to predict both short-term and long-term academic success, positive interpersonal relationships, and overall developmental trajectories (Murano et al., 2020). According to social learning theory, children attain social functioning and adjustment by observing and imitating significant others in their daily interactions, particularly within the context of close relationships, such as the parent–child bond (Rumjaun & Narod, 2020). The extent to which parents engage with their children’s lives and establish connections is a reflection of their parenting beliefs or attitudes towards child-rearing and education. Developmentally appropriate and child-centered beliefs can potentially act as catalysts, aiding parents to adopt effective parenting behaviors that promote their children’s development (L. Ren et al., 2024). However, in complex contexts, parenting beliefs may not always translate directly into their practices with fidelity (Bornstein et al., 2018), and time spent with children’s lives may not necessarily foster a positive parent–child relationship characterized by warmth, sensitivity, and responsiveness (Palkovitz, 2019). Thus, it is crucial to gain a deeper understanding of the cascade from parenting beliefs to practices, to relationships, and ultimately to children’s social adjustment.

The present study focuses on the paternal side of parenting. Much of the existing literature concerning parenting mechanisms has primarily centered on mothers or has merged parental information, with relatively few investigations dedicated to pathways within the fathering subsystem (Fortunato et al., 2025; Wu et al., 2023). It is necessary to gain insights into paternal mechanisms when considering recent evidence underscoring the uniqueness of the father–child relationship (Almeida et al., 2025). This is particularly relevant in contemporary China, where social transformations are encouraging fathers to move beyond traditional Confucian patriarchal roles toward greater emotional and practical engagement in young children’s lives (Li, 2020).

Grounded in family systems theory, which posits that each family subsystem can have its own dynamics, this study aims to test the associations between Chinese fathers’ beliefs, involvement, relationships with their children, and their children’s social adjustment. By recognizing fathers as a meaningful source of influence within the web of family dynamics, this research seeks to delineate the mechanisms through which paternal factors shape child outcomes, thereby contributing to a comprehensive understanding of the family processes.

### 1.1. Literature Review

#### 1.1.1. Theoretical Framework

The theoretical framework of this study is rooted in the following two complementary foundations: family systems theory (Cox & Paley, 1997) and an extended model of Bornstein et al.’s (2018) “beliefs-practices-outcome” framework by incorporating the father–child relationship as a key mediator. Family systems theory justifies our focus on the father–child subsystem: it posits the family as a complex, interactive unit of interdependent yet distinct subsystems whose internal processes and dynamics can be examined independently. Accordingly, this study centers on the father–child subsystem to investigate its specific mechanisms, namely, how paternal factors are linked to child outcomes within this dyad.

Building on this subsystem focus, we extended Bornstein et al.’s (2018) original “beliefs-practices-outcomes” model by adding the father–child relationship as a critical mediating variable. Developmentally appropriate paternal beliefs lay the foundation for parenting practices, which, in turn, shape child outcomes (Bornstein et al., 2018). However, the father–child relationship, which is cultivated through ongoing interactions, acts as a crucial bridge linking specific parenting practices to child adjustment. While the existing studies have explored the model linking parenting beliefs, practices, and child outcomes (L. Ren et al., 2024; Zhong et al., 2020), there is a gap in examining the father–child relationship as a mediating mechanism. By integrating family systems theory with this extended “beliefs-practices-relationships-outcomes” model, this study aims to explore a sequential chain: from paternal beliefs and involvement to the father–child relationship, and finally to child adjustment within the father–child subsystem.

#### 1.1.2. Relationship Between Fathers’ Belief and Child’s Social Adjustment

While a myriad of the existing literature has extensively explored the role of parenting practices in child development (e.g., Cumming et al., 2022; Marcone et al., 2021), researchers have largely overlooked empirical explorations on the relations between parenting beliefs and child outcomes, despite the early awareness that parental beliefs regarding child-rearing, adult authority, and parenting roles can affect every aspect of children’s development (Miller, 1995). Schaefer and Edgerton (1985) conceptualized parents’ beliefs about children as a spectrum, with progressive, child-centered views on one end, and traditional, adult-centered beliefs on the other. Progressive parents typically respect children’s independence and autonomy, and they support children to engage in self-directed activities. Counter to this, traditional parents emphasize children’s obedience to adults, as well as strict discipline and control in upbringing (L. Ren & Pope Edwards, 2015). In this study, fathers’ progressive beliefs refers to an orientation that emphasize encouraging children’s autonomy, exploration, and challenge-taking within a supportive environment.

Scattered evidence could lend credence to an expected connection between fathers’ beliefs and children’s social adjustment. For example, J. Shears and Robinson (2005) found that fathers who held progressive beliefs tended to have toddlers with higher mental development and fewer behavioral problems. This was because fathers of this kind allowed more exploratory opportunities and were less controlling of their children’s social activities. Conversely, traditional parents exhibited higher problem behaviors in later childhood (Correa Ramirez, 2024). Notably, caregivers’ beliefs appear more strongly predictive of children’s social–emotional development than other early developmental outcomes, such as cognition, language, and motor skills (Zhong et al., 2020). This may be attributed to the malleability of social–emotional competence during early childhood (Francesconi & Heckman, 2016).

#### 1.1.3. The Mediating Role of Father Involvement

Father involvement encompasses the extent of the fathers’ direct and indirect engagement in their children’s lives, including emotional support, financial contributions, and caregiving responsibilities (Lamb, 2000). Over the past two decades, researchers have increasingly recognized the substantial implications of father involvement for various domains, such as child development, marital quality, and family well-being (Almeida et al., 2025; Diniz et al., 2021; Volling & Bornstein, 2025).

Father involvement is affected by a range of factors, such as socioeconomic status and child characteristics, to name two (Saifuddin & Ezzi, 2024). Notably, the role of fathers’ child-rearing beliefs, which is a crucial internal factor, has been relatively overlooked (Correa Ramirez, 2024; Y. Liu et al., 2022). The well-established “belief-practice-outcome” model suggests that parental practices reflect underlying beliefs (L. Ren & Pope Edwards, 2015). To illustrate, Bornstein et al. (2018) conducted a longitudinal study over eight years, revealing that parenting cognition during infancy, particularly at 20 months, predicted nurturing parenting styles by the age of 4, which, in turn, predicted a reduction in anger–aggression behaviors by the age of 10. This suggests that a similar pathway may exist for fathers: fathers’ involvement practices may reflect their beliefs about fostering children’s social competence and behavioral adaptability.

This study also considered mother-reported father involvement to offer a broader perspective and to mitigate the potential single-informant biases. Previous research has unveiled that both father and mother reports of father involvement are reliable, while noting that discrepancies can occur (Hernandez & Coley, 2007). This discrepancy accentuates the importance of a multi-informant assessment of father involvement.

#### 1.1.4. The Mediating Role of the Father–Child Relationship

The father–child relationship is conceptually and operationally anchored in Pianta’s (1992) relational model of early childhood parent–child interactions, which posits that the father–child relationship is captured by the following two independent dimensions (Driscoll & Pianta, 2011): closeness and conflict. Father–child closeness refers to the affective warmth and positive emotional bonding in the relationship; whereas, father–child conflict points to the extent of negativity and confrontations between fathers and their children (Zhang, 2013). These two dimensions are separate, albeit related: the absence of conflict does not automatically imply a close bond, and vice versa (Driscoll & Pianta, 2011). This suggests the necessity of examining these dimensions separately when assessing children’s developmental outcomes (Lang et al., 2020). Unlike attachment (Bowlby, 1969) or general parenting quality (Baumrind, 1971), the father–child relationship reflects dyadic, observable, daily interaction patterns rather than children’s internal working models or stable caregiver practices.

The development of children’s social skills, especially during early childhood, is profoundly tied to the relationships they form with their parents (Hosseini et al., 2022). Fathers may influence their children’s social competence through specific relationship-related mechanisms (Fernandes et al., 2020). For example, fathers often engage in physically interactive play with their children, which can be characterized by high energy and a focus on excitement (Teufl & Ahnert, 2022). Such interactions can provide opportunities for children to practice emotional regulation and behavioral competencies. In spite of the relative dearth of studies in China, Zhang (2012, 2013) has highlighted the role of the father–child relationship in children’s early social adjustment.

Although the precursor roles of parenting beliefs and the parent–child relationship in influencing children are acknowledged, the empirical evidence linking these constructs remains very limited. Parenting beliefs are likely to influence their interactions with children and the relationships that develop as a result. Therefore, it is plausible that paternal beliefs may function on child social adjustment indirectly through the father–child relationship.

#### 1.1.5. The Chain Mediating Roles of Father Involvement and the Father–Child Relationship

This study examined both father involvement and the father–child relationship act as sequential mediators in the pathway from fathers’ progressive beliefs to children’s social adjustment. This hypothesis draws on the ‘beliefs-practices-outcomes’ model, which posits that fathers’ beliefs shape their level of involvement, which, in due course, links to the father–child relationship, and ultimately impacts child outcomes (Finardi et al., 2022).

Despite a robust theoretical foundation, few empirical research has tested this integrated pathway among fathers (Bornstein et al., 2018; L. Ren et al., 2024). Moreover, father involvement and the father–child relationship, though closely linked, are not identical. Children can benefit from a strong father–child relationship even when direct involvement is limited, suggesting that the close bonding within the relationship may play a more proximal role in children’s adjustment (Jessee & Adamsons, 2018). Hence, it can be inferred that these two constructs may play distinct yet connected mediating roles between fathers’ beliefs and children’s outcomes.

#### 1.1.6. The Present Study

The current study aimed to advance the literature by exploring the sequential mediating roles of father involvement and the father–child relationship in the link between paternal beliefs and children’s social adjustment within Chinese families. The theoretical models and empirical linkages among these constructs have been primarily established within Western cultural contexts, highlighting a gap in the understanding of fathering dynamics within the specific sociocultural setting of China (Zheng et al., 2026). Traditionally, Chinese fathers have been primarily viewed as breadwinners, while child-rearing and education have been predominantly in the mother’s domain. However, as women’s roles and social status have expanded beyond the household, fathers are expected to become more involved in child-rearing and education (Lin et al., 2020). Concurrently, Chinese parents have historically emphasized academic achievement within a highly competitive societal framework, while simultaneously adopting progressive beliefs that highlight holistic child development, including social competence, influenced by Western parenting ideologies (Y. Liu et al., 2021). This evolving context makes the study of fathering in China a valuable and timely endeavor.

This study seeks to address the following research questions:

1. To what extent are fathers’ progressive beliefs about children associated with children’s social adjustment?
2. To what extent do father involvement and the father–child relationship (two dimensions: conflict and closeness) mediate the association between fathers’ progressive beliefs and children’s social adjustment?

Guided by the prior literature review, we proposed the following hypotheses to answer these research questions.

**H1.**  *Fathers’ progressive beliefs about children are positively related to children’s social adjustment.*

**H2.**  *Fathers* *’ progressive beliefs about children are positively related to father involvement; greater father involvement is associated with better social adjustment.*

**H3.**  *Father–child closeness mediates the relationship between fathers’ progressive beliefs about children and children’s social adjustment. Specifically, stronger progressive beliefs are associated with higher levels of father–child closeness; higher closeness is associated with better social adjustment.*

**H4.**  *Father–child conflict mediates the relationship between fathers’ progressive beliefs about children and children’s adjustment. Specifically, stronger progressive beliefs are associated with lower levels of father–child conflict; lower conflict is associated with better social adjustment.*

**H5.**  *Father involvement and the father–child relationship serve as a chain mediating role in the relationship between fathers’ progressive beliefs about children and children’s social adjustment. We hypothesize a positive serial mediation pathway: stronger progressive beliefs are associated with greater father involvement; greater father involvement is associated with higher closeness and/or lower conflict, which, in turn, is associated with better social adjustment.*

**Figure 1:**
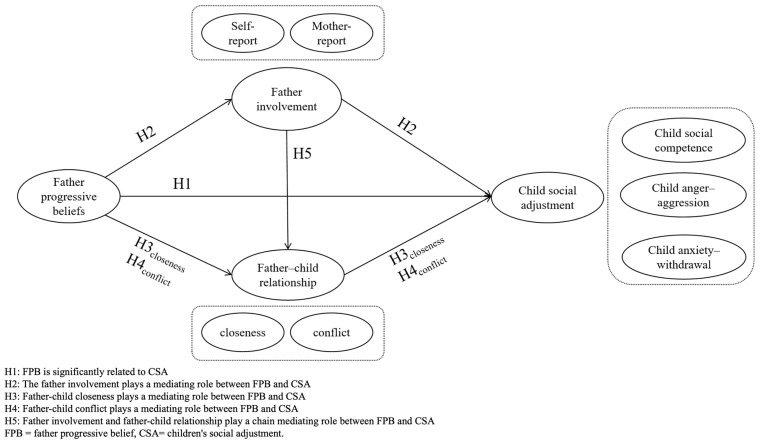
The Hypothesized Model.

The hypothesized model can be seen in Figure 1.

## 2. Method

### 2.1. Participants

Participants in this study included 1862 mother–father dyads (i.e., 1862 mothers and 1862 fathers) aged between 18 and 63 years from Guangdong Province, southeastern China. Parents provided information about their children aged between 28 and 82 months (*M* = 55.25, *SD* = 10.53). The sample comprised families from various regions within Guangdong. Of these families, 60.3% had two children, 28.6% had one child, and 11.1% had three or more children. The family socioeconomic status (SES) was calculated following C. Ren (2010). A factor analysis incorporating six indicators—highest education levels, occupations, and annual incomes of both mothers and fathers—revealed a single factor with an Eigenvalue greater than one, explaining 46.65% of the variance. The SES was computed as follows: SES = (0.79 × mother’s education level + 0.62 × mother’s annual income + 0.73 × mother’s occupation + 0.75 × father’s education level + 0.45 × father’s annual income + 0.70 × father’s occupation)/0.47. The resulting SES distribution denoted that 25.7% of the families were classified as low-income, 47.7% as middle-income, 20.2% as high-income, and 6.3% of the families did not report this. The demographic statistics are presented in Table 1.

### 2.2. Procedure

This study received ethical approval from the Ethics Review Board of South China Normal University and was conducted in accordance with the Declaration of Helsinki. Participants were recruited using stratified sampling. First, regions representing low, medium, and high economic development levels in Guangdong Province were identified in 2022. Within each region, 10–18 preschools (38 total) were randomly selected based on funding source (public/private) and geographic location (urban/rural). From each preschool, two classrooms per grade level (first, second, and third/last year, serving children aged 3, 4, and 5, respectively) were randomly chosen, and approximately eight preschoolers per classroom were randomly recruited, ensuring sample representativeness. The final sample comprised 1862 mother–father dyads (3724 participants).

Data were collected over two months (October–November 2023) by eight trained research assistants closely supervised by the authors. Preschools were approached through personal networks with assistance from the local department of education. Following school agreement, parents received an information letter and could opt out. Both parents independently completed an online questionnaire in about 15 min (Wenjuanxing platform) covering the study variables. Schools did not collect any information linking individual children to specific classes on the questionnaires.

### 2.3. Measures

Father Progressive Beliefs. Fathers reported their progressive beliefs about children using the progressive belief sub-scale of the Parental Modernity Scale (Schaefer & Edgerton, 1985), which treats adults’ beliefs about children as unidimensional constructs. We adopted the Chinese version of this scale validated by B. Y. Hu et al. (2023). This version has been extensively used in prior research with Chinese populations, attesting to its utility and acceptance (L. Ren et al., 2024). It was developed following established cross-cultural adaptation guidelines, including independent forward- and back-translation steps, to ensure semantic and conceptual equivalence with the original measure. Eight items asking about the fathers’ progressive beliefs were used to measure the fathers’ progressive beliefs. Higher scores reflect stronger progressive beliefs, indicating that fathers with more progressive views endorsed these items to a greater extent (e.g., “Children can disagree with their parents”). In a confirmatory factor analysis, three items had negative loadings (e.g., “When children are playing role games, parents should support and cooperate with them”). As these items appear to focus more on reflections of the roles of parents than the child, we excluded these three items. The fathers responded on a five-point scale, ranging from 1 (Never) to 5 (Very frequent). In the present study, Cronbach’s alpha for a father’s progressive belief was .69.

Father Involvement. Both fathers and mothers reported on the fathers’ parenting participation over the past year. We employed the Inventory of Father Involvement Questionnaire (Hawkins et al., 2002), a tool specifically developed to assess fathers’ involvement in children’s lives, to precisely capture the fathers’ parenting practices. This scale has been validated for adaptation to the Chinese social context by Yin et al. (2012). For the mother-reported version, we made a slight modification to the statements by changing the subject from “you” to “he”. The scale contains 26 items and four dimensions, including support and planning, daily care, encouragement and praise, and discipline and teaching responsibility. Example items include, among others, “You/He disciplines the child”. Participants answered the items on a five-point Likert scale, ranging from 1 (Never) to 5 (Always). Higher scores indicate greater levels of fathers’ parenting participation. In the present study, Cronbach’s alphas were .80/.76 for discipline and teaching responsibility, .91/.91 for support and planning, .89/.89 for daily care, and .82/.80 for encouragement and praise, for the father and mother reports, respectively. In the mediation analysis, we used the mean of each dimension as indicators for the latent variable of father involvement.

Mother Involvement. Since there is no parallel measure of paternal involvement among mothers, we selected another widely used scale, the Family Involvement Questionnaire–Short Form (Fantuzzo et al., 2013), to evaluate mother involvement in children’s lives. This scale has been validated in the Chinese context according to rigorous validation process (Q. Q. Liu & Li, 2019). This scale consists of 21 items and three dimensions, including parent–teacher interactions, school involvement, and family involvement. Example items, among others, include “I talk to my child’s teacher about his/her daily school routine”. Mothers answered the items on a five-point scale, ranging from 1 (Never) to 5 (Always). Higher scores indicate greater levels of mothers’ parenting participation. In the present study, Cronbach’s alphas were .91 for parent–teacher interactions, .87 for school involvement, and .86 for family involvement.

Father–Child Relationship. A widely used measure was used for fathers to report their perceptions of closeness and conflicts with their children (Pianta, 1992). This scale has been validated and widely used in the Chinese context (Zhang et al., 2008). There are 7 items for closeness (e.g., “I share an affectionate, warm relationship with my child”) and 8 items for conflicts (e.g., “My child is uncomfortable with physical affection or touch from me”). Fathers responded on a five-point scale, ranging from 1 (Never) to 5 (Always). Higher scores indicate greater levels of fathers’ perceptions of closeness or conflicts with their children. In the present study, Cronbach’s alphas were .88 for closeness and .89 for conflicts.

Child Social Adjustment. Fathers reported their children’s social competence, anxiety–withdrawal behavior, and anger–aggression behavior using the short version of the Social Competence and Behavior Evaluation Scale (LaFreniere & Dumas, 1996). We also used the Chinese version which was validated by Y. Liu et al. (2012) following a standard procedure. There are 30 items in total with three dimensions, including Anger–aggression (e.g., “Hits teacher or destroys things when angry with teacher”; 9 items), Anxiety–withdrawal (e.g., “Doesn’t talk or interact during group activities”; 11 items), and Social competence (e.g., “Takes other children and their point of view into account”; 10 items). Fathers responded on a five-point scale, ranging from 1 (Never) to 5 (Very frequent). Higher scores indicate greater social competence as well as higher levels of internalizing and externalizing behavior problems. In the present study, Cronbach’s alphas were .88 for anger–aggression, .91 for anxiety–withdrawal, and .86 for social competence.

### 2.4. Data Analyses

Prior to the main analyses, the raw data were systematically cleaned. Missing values were minimal due to mandatory completion of the online platform. One case with an unrealistic child age was identified and treated as missing. Missing data were handled using the Full Information Maximum Likelihood (FIML) estimation in *M*plus under the missing-at-random assumption, which yields unbiased estimates even with minimal missing data (Enders, 2001).

Formal analyses were conducted in *M*plus 8.8 (Muthén & Muthén, 1998–2012). Two chain mediation models were tested: Model 1 used the father-reported data; Model 2 substituted the mother-reported father involvement for the father-reported involvement. Modification indices would be reviewed for poor-fitting models to add consistent residual correlations. All models controlled for the child’s age, gender, family SES, and maternal involvement. A sensitivity analysis was conducted on the child age. Because social competence matures with age, models with and without child age as a covariate were compared to ensure the pathways were not artifacts of developmental changes; no significant differences would suggest age-related maturation did not affect outcomes.

Model fit was assessed using the chi-squared statistics (*χ*^2^), root mean square error of approximation (RMSEA), comparative fit index (CFI), and standardized root mean square residual (SRMR; Asparouhov & Muthén, 2018). RMSEA and SRMR values below 0.08 and CFI values above 0.90 indicated acceptable fit (L. Hu & Bentler, 1999). Nested models were compared using the Satorra–Bentler corrected chi-squared difference tests (Satorra & Bentler, 2010). Data are available upon reasonable request.

## 3. Results

To assess common method bias, Harman’s single-factor test was conducted. All self-reported items were entered into an unrotated exploratory factor analysis with extraction fixed to one factor. A single factor accounted for 17.43% of the total variance, below the conventional 40% threshold (Aguirre-Urreta & Hu, 2019), indicating that common method bias was not a substantial concern. Due to the hierarchical structure of our data, we further assessed clustering at the preschool level by calculating intraclass correlation coefficients (ICCs) for all key variables to see if multilevel modeling was necessary. The results showed that the ICC values ranged from 0.02% to 3.40%, with the vast majority below 2%. These values are generally considered negligible and not problematic for biasing standard errors in multilevel contexts.

Table 2 presents the descriptive statistics, skewness, kurtosis, and correlations among the observed variables. The skewness and kurtosis values for all study variables were within acceptable ranges (skewness < |3|, kurtosis < |10|; Kline, 2011), suggesting that the data were normally distributed. Most correlations were as anticipated, with two exceptions: fathers’ progressive beliefs did not significantly correlate with either aspect of problem behaviors; additionally, mother-reported father involvement showed correlations similar to father-reported involvement, except for father–child conflict. Mother-reported involvement negatively correlated with father–child conflict, whereas father-reported involvement did not.

**Figure 2:**
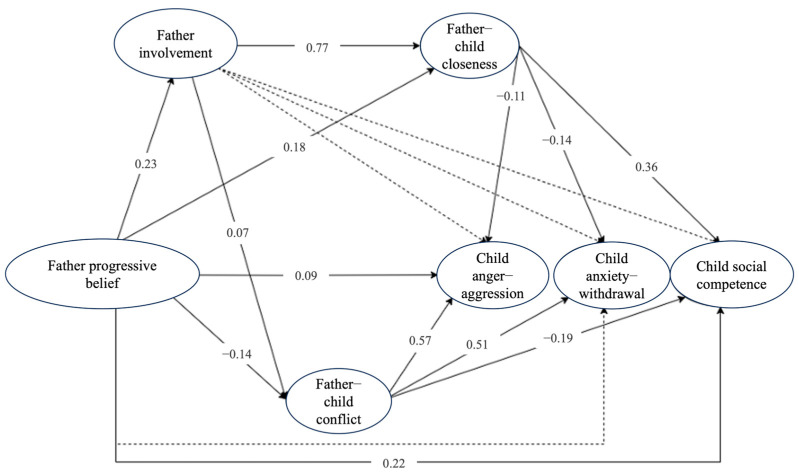
Standardized Effects in Model 1 with Father Reports. *Notes*: These are standardized model 1 results. The dotted lines show nonsignificant effects.

The model based on father reports was not optimal, *χ*^2^ (1306) = 5985.55, CFI = 0.900, RMSEA = 0.044, 90% CI [0.043, 0.045], SRMR = 0.045. Similarly, the model with mother-reported father involvement also showed suboptimal fit, *χ*^2^ (1306) = 5888.65, CFI = 0.898, RMSEA = 0.043, 90% CI [0.042, 0.045], SRMR = 0.044. To improve model fit and to maintain consistency, we reviewed the modification indices for both models. Items 1 and 5 in the father beliefs scale (i.e., “Because parents lack professional education training, it is not appropriate to question the teaching methods of teachers” and “Children will not do the right thing unless they must”) and items 1 and 2 in the parent–child conflicts scale (i.e., “My child and I always seem to be struggling with each other” and “My child is uncomfortable with physical affection or touch from me”) had the highest residual correlations. Adding these residual correlations to both models resulted in improved fit: the mother-reported model yielded *χ*^2^ (1304) = 5581.53, CFI = 0.905, RMSEA = 0.042, 90% CI [0.041, 0.043], SRMR = 0.043, and the father-reported model showed *χ*^2^ (1304) = 5677.86, CFI = 0.907, RMSEA = 0.042, 90% CI [0.041, 0.044], SRMR = 0.044. Table 3 and Figure 1 and Figure 2 display the effects from these models.

**Figure 3:**
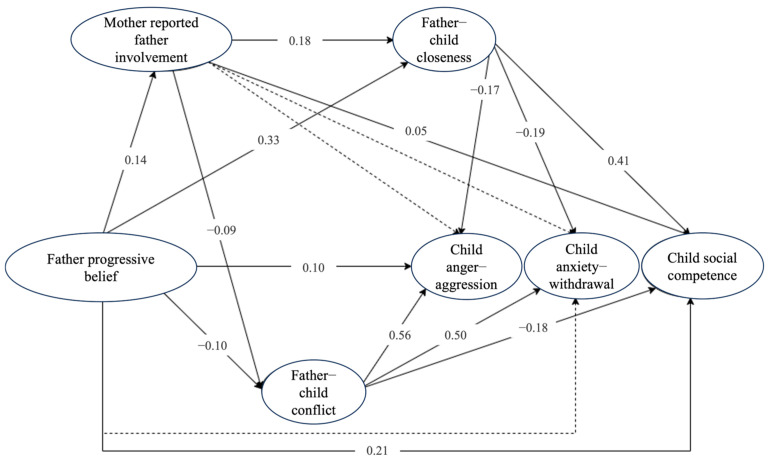
Standardized Effects in Model 2 with Mother-Reported Father Involvement. *Notes*: These are standardized model 2 results. The dotted lines show nonsignificant effects.

The results of the chain mediation models, controlling for the child’s age (grade), gender, family SES, and maternal involvement, are presented in Table 3 and Figure 2 and Figure 3. A sensitivity analysis indicated that the results remained consistent regardless of whether the child age was included as a covariate or whether the single case with missing age data was excluded, confirming the robustness of the core findings. The analyses revealed several significant indirect pathways with small to moderate effects linking fathers’ progressive beliefs to child outcomes through father involvement and the father–child relationship; however, the effects varied by outcome and relationship dimension.

Figure 2 illustrates the standardized effects in mediation Model 1 with father-reported father involvement. The chain effect of fathers’ progressive beliefs on child social competence through father involvement and father–child closeness was significant and positive, β = 0.07, 95% CI [0.044, 0.093]. Conversely, the chain effect through father involvement and father–child conflict was significant and negative, *β* = −0.003, 95% CI [−0.007, −0.001]. The direct effect of fathers’ progressive beliefs on social competence was positive, *β* = 0.22, 95% CI [0.149, 0.291]. Progressive beliefs positively correlated with higher father involvement, which, in turn, was positively associated with father–child closeness and higher child social competence. However, while progressive beliefs positively correlated with higher father involvement, higher involvement was negatively associated with father–child conflict and lower child social competence.

For child anger–aggression, the chain effect through father involvement and father–child closeness was significant and negative, *β* = −0.04, *p* = .002, whereas the direct effect was not, *β* = 0.04, *p* = .374, suggesting full mediation. By contrast, the chain effect through father involvement and father–child conflict was significant and positive, *β* = 0.01, *p* = .024, alongside a significant direct effect, *β* = 0.08, *p* = .027. Overall, the indirect effects were *β* = −0.02, 95% CI [−0.042, −0.004] for closeness and *β* = 0.01, 95% CI [0.003, 0.019] for conflict, with a total direct effect of *β* = 0.09, 95% CI [0.025, 0.159].

For child anxiety–withdrawal, the chain effect through father involvement and father–child closeness was significant and negative, *β* = −0.02, 95% CI [−0.046, −0.009], whereas through father involvement and father–child conflict was significant and positive, *β* = 0.01, 95% CI [0.002, 0.017]. The direct effect was not significant, *β* = 0.01, *p* = .723, indicating that the association is primarily explained by father involvement and father–child relationships.

Additional simple mediation effects through the father–child relationship only were also observed. Fathers’ progressive beliefs indirectly predicted child social competence via father–child closeness, *β* = 0.06, 95% CI [0.043, 0.087], and via father–child conflict, *β* = 0.03, 95% CI [0.012, 0.046]. For child anger–aggression, the indirect effects were *β* = −0.02, 95% CI [−0.041, −0.004] through closeness and *β* = −0.08, 95% CI [−0.124, −0.040] through conflict. For child anxiety–withdrawal, the indirect effects were *β* = −0.02, 95% CI [−0.046, −0.007] through closeness and *β* = −0.07, 95% CI [−0.109, −0.035] through conflict.

The Model 2 (mother-reported father involvement) results were largely consistent with Model 1, with three exceptions. First, the indirect effect of fathers’ progressive beliefs on child social competence through mother-reported father involvement was significant and positive, *β* = 0.01, 95% CI [0.001, 0.015]. Second, unlike Model 1, the effect of mother-reported father involvement on father–child conflict was negative, *β* = −0.09, 95% CI [−0.138, −0.038]. Third, the effect on father–child closeness was weaker than in Model 1, *β* = 0.18, 95% CI [0.119, 0.237].

## 4. Discussion

Grounded in family systems theory and the extended “parenting beliefs → parenting practices → parent–child relationship → child development” conceptual model, and rooted within the Chinese cultural context, this study identified a consistent sequential mediation mechanism of father involvement and the father–child relationship, which explained how paternal beliefs transmit to both positive and negative indicators of children’s social adjustment through behavioral and relational pathways.

### 4.1. The Dual Pathways from Paternal Beliefs to Children’s Social Adjustment

This study revealed that fathers’ progressive beliefs are directly linked to children’s social competence and anger–aggression, which partially supports Hypothesis 1 (Zhong et al., 2020). In addition, father involvement and the father–child relationship were identified as sequential mediators in these links, providing support for Hypotheses 3, 4, and 5 (L. Ren et al., 2024). Notably, the findings point to the complex dual pathways within these sequential mediation mechanisms, thus partially supporting Hypotheses 2 and 5. Specifically, paternal beliefs were associated with higher levels of father involvement, which, in turn, brought two distinct relational outcomes: greater father–child closeness and increased father–child conflict (though the latter effect is very weak yet statistically significant). These two relational pathways then exerted opposing effects on the children: closeness was associated with better social adjustment, while conflict was associated with weaker adjustment, with conflict being a stronger predictor of problem behaviors. These highlighted that the mediated associations are fundamentally shaped by the father–child relationship.

The observed contradictory pathway may reveal culturally specific patterns that both converge with and diverge from Western research on father involvement. Consistent with Western literature (Correa Ramirez, 2024), progressive beliefs were found to motivate Chinese fathers to increase child-rearing involvement, supporting the universal proposition that parenting beliefs shape parenting behaviors (L. Ren et al., 2024). However, unlike the predominantly positive paternal involvement–child adjustment links reported in Western studies (Diniz et al., 2021), our results uncover a dual pathway in the Chinese context: suboptimal or low-quality paternal involvement that triggers parent–child conflict undermines children’s social adjustment.

This conflict pathway reflects China’s transitional cultural context. Traditionally, fathers were positioned as disciplinary figures with limited caregiving roles and emotional communication with children, but modern ideologies now emphasize paternal involvement and child-centered parenting, fundamentally shifting societal expectations of fatherhood. Yet, new values coexist with insufficient support for fathers to translate these beliefs into high-quality, conflict-free involvement (L. Ren et al., 2024), resulting in diverse and sometimes contradictory parenting practices (Lu et al., 2024). Specifically, increased but inappropriate paternal involvement, such as a habitual high-control parenting style (Ng et al., 2007), may trigger conflict. This mismatch between progressive beliefs and underdeveloped parenting competence explains why greater involvement is concurrently associated with closer bonds and more conflict. This finding aligns with prior research documenting the complexities of implementing parenting practices in rapidly changing social environments (Palkovitz, 2019) and underscores the necessity of situating parenting processes within their specific cultural contexts.

### 4.2. The Differences Between Self-Reported and Mother-Reported Father Involvement

This study also revealed differences between fathers’ self-reports and mothers’ reports of paternal involvement. Consistent with previous research (Charles et al., 2018), fathers rated their own involvement higher than did mothers. Our findings further showed that mother-reported father involvement was positively related to father–child closeness and negatively related to father–child conflict. From the mothers’ perspective, increased father involvement correlates with a closer and less conflicting relationship. On the contrary, father-reported involvement was positively associated with both closeness and conflict. In addition, fathers’ perception of their own involvement did not mediate the link between their beliefs and child social adjustment; instead, the pathway went through the father–child relationship. By contrast, mothers perceived father involvement itself as a significant mediator.

These discrepant reports may be because of perspectives and value differences between mothers and fathers. Mothers, often acting as keen observers and managers of family interactions, may place greater weight on tangible, observable engagement activities as direct indicators of positive father contribution (Wang & Schoppe-Sullivan, 2021). For fathers, the emotional tone of interactions may be more salient than the mere frequency of acts. Therefore, while mothers may view increased father–child interactions as reducing conflict and increasing closeness, fathers might experience clashes due to inappropriate or incompatible involvement styles. Such perceptual differences help explain the informant discrepancy observed in this study. This underscores the value of incorporating multiple informants of father involvement to gain a more comprehensive picture of family dynamics.

Collectively, the current study, focusing on the father–child subsystem, advances the literature by exploring the serial mediating mechanisms of practices and relationships linking these beliefs to child outcomes. In other words, considering how beliefs translate into practices, and in particular, how practices shape relationship during the parenting process are important.

### 4.3. Limitations

Several limitations of the present study must be considered. First, the primary limitation stems from the research design. While we controlled for a global measure of maternal involvement, it did not incorporate parallel measures of maternal beliefs, mother–child relationships, or other key maternal factors. Therefore, the observed pathways cannot be interpreted as evidence of effects unique to fathers. We can only conclude that paternal factors correlate with child outcomes in a theoretically coherent way, without confirming distinct paternal mechanism. Second, the cross-sectional design precludes causal relationships. The present study also did not examine potential moderators that might influence the serial models, particularly the complex links between father involvement and father–child relationships. Third, relying solely on parent reports introduces potential common method bias. Despite acceptable Harman’s single-factor test results, future studies should incorporate multi-method assessments (e.g., observation).

## 5. Conclusions and Implications

This study makes two key theoretical contributions to the parenting and child development literature. First, it extends the classic “beliefs-practices-outcomes” framework (Bornstein et al., 2018) by empirically validating the father–child relationship as a critical proximal mediator. While prior research has established the links between parenting beliefs, practices, and child outcomes, the present findings clarify that the indirect effects of fathers’ beliefs on child social adjustment are primarily mediated through the father–child relationship, with smaller effects mediated by father involvement. This refinement helps explain why greater paternal involvement does not always translate into better child outcomes (Zheng et al., 2026), highlighting the father–child relationship as a key boundary condition within the family system. Second, this study provides culturally grounded evidence from mainland China, addressing the overreliance on Western samples in parenting research. By analyzing parenting processes within China’s ongoing social and cultural transition, it challenges the unidimensional view of parenting beliefs and involvement, and reveals the paradoxical nature of contemporary fathering. By doing so, it expands the cross-cultural generalizability of parenting theories and offers a culturally sensitive perspective for understanding fatherhood in non-Western contexts.

Practically, this study has significant implications for family education and parenting programs in China. Initiatives aimed at supporting child social adjustment could take the father–child subsystem into consideration. Rather than simply focusing on whether fathers are involved in their children’s lives, it is important for practitioners to pay closer attention to the specific approaches Chinese fathers adopt when participating in their children’s upbringing (i.e., positive relationship-building parenting practices). In the Chinese context, simply encouraging fathers to take part in child-rearing is not sufficient on its own. It is equally essential to guide them on the appropriate ways to engage, which can effectively minimize conflicts stemming from improper discipline. This focus on the interaction quality, as opposed to merely the quantity, is crucial. Even relatively infrequent direct involvement can benefit children, if accompanied by a strong emotional, supportive father–child bond (Jessee & Adamsons, 2018). Thus, efforts that help fathers foster warm, low-conflict relationships with their children may be more beneficial. In addition, the above initiatives should be framed within the context of supporting the entire family system. Our goal is not to elevate the paternal role above others, but to recognize and support the contributions of all family members.

## Figures and Tables

**Table 1:** Demographic Statistics for Child and Family Demographic Variables (*N* = 1862 Father–Mother Dyads).

Demographic Variable	*M* ± *SD*	Category	*N* (Percentage)
Child age (months)	55.25 ± 10.53		
Grade (3-, 4-, 5-year-olds)		First year	588 (31.6%)
		Second year	648 (34.8%)
		Third year	625 (33.6%)
		Missing	1 (0.1%)
Father age (years)	35.78 ± 5.45		
Mother age (years)	33.84 ± 4.89		
Child Gender		Male	1048 (56.3%)
		Female	814 (43.7%)
Family Socioeconomic Status		Low	479 (25.7%)
		Middle	888 (47.7%)
		High	377 (20.2%)
		Missing	118 (6.3%)
Number of children in a family		1	533 (28.6%)
		2	1122 (60.3%)
		>2	207 (11.1%)

*Note*. *M* = mean; *SD* = standard deviation. Continuous variables are reported as *M* ± *SD*; categorical variables are reported as *N* (percentage).

**Table 2:** Descriptive and Zero-Order Bivariate Correlations Between the Study variables.

	Mean	*SD*	1	2	3	4	5	6	7	8
1. FI	4.32	.58	-							
2. Mother-reported FI	4.24	.62	.25 *	-						
3. FCCLS	4.29	.61	.74 *	.21 **	-					
4. FCCOT	2.64	.95	.04	−.10 **	−.01	-				
5. CSC	3.56	.58	.37 *	.17 **	.45 **	−.21 **	-			
6. CAA	2.31	.65	−.12 *	−.09 **	−.15 **	.49 **	−.14 **	-		
7. CAW	2.21	.66	−.15 *	−.11 **	−.19 **	.48 **	−.23 **	.73 **	-	
8. FPB	3.81	.61	.17 **	.12 **	.24 **	−.05 *	.28 **	.04	−.03	-
Skewness		0.06	−2.09	−1.01	−1.42	0.51	−0.04	0.70	0.83	−0.17
Kurtosis		0.11	7.98	1.28	4.60	−0.11	1.17	1.47	1.80	0.17

*Notes*. *SD* = standard deviations. ** *p* < .01, * *p* < .05. FI = father involvement, FCCLS = father–child closeness, FCCOT = father–child conflict, CSC = child social competence, CAA = child anger–aggression, CAW = child anxiety–withdrawal, FPB = father progressive belief.

**Table 3:** Standardized Direct and Indirect Effects in the Father-Reported Model 1 and the Mother-Reported Model 2.

Paths	Model 1				Model 2			
	*β*	*SE*	95% CI		*β*	*SE*	95% CI	
			LLCI	ULCI			LLCI	ULCI
*Chain effects*								
C1a: FPB → FI → FCCLS → CSC	0.07 *	0.01	.044	.093	0.01 *	0.003	.005	.016
C1b: FPB → FI → FCCOT → CSC	−0.003 *	0.001	−.007	−.001	0.002 *	0.001	.001	.004
C2a: FPB → FI → FCCLS → CAA	−0.02 *	0.01	−.042	−.004	−0.004 *	0.001	−.007	−.002
C2b: FPB → FI → FCCOT → CAA	0.01 *	0.004	.003	.019	−0.01 *	0.002	−.012	−.003
C3a: FPB → FI → FCCLS → CAW	−0.02 *	0.01	−.046	−.009	−0.004 *	0.001	−.008	−.003
C3b: FPB → FI → FCCOT → CAW	0.01 *	0.004	.002	.017	−0.01 *	0.002	−.011	−.003
*Other indirect effects*								
Ind1a: FPB → FI → CSC	0.02	0.01	−.004	.039	0.01 *	0.004	.001	.015
Ind1b: FPB → FI → CAA	−0.02	0.01	−.043	.001	−0.003	0.003	−.011	.002
Ind1c: FPB → FI → CAW	−0.02	0.01	−.043	.002	−0.01	0.004	−.014	.000
Ind2a: FPB → FCCLS → CSC	0.06 *	0.01	.043	.087	0.13 *	0.02	.106	.164
Ind2b: FPB → FCCLS → CAA	−0.02 *	0.01	−.041	−.004	−0.06 *	0.01	−.081	−.033
Ind2c: FPB → FCCLS → CAW	−0.02 *	0.01	−.046	−.007	−0.06 *	0.01	−.085	−.042
Ind3a: FPB → FCCOT → CSC	0.03 *	0.01	.012	.046	0.02 *	0.01	.006	.034
Ind3b: FPB → FCCOT → CAA	−0.08 *	0.02	−.124	−.040	−0.06 *	0.02	−.099	−.017
Ind3c: FPB → FCCOT → CAW	−0.07 *	0.02	−.109	−.035	−0.05 *	0.02	−.088	−.016
*Direct effects*								
D1: FPB → CSC	0.22 *	0.04	.149	.291	0.21 *	0.04	.143	.286
D2: FPB → CAA	0.09 *	0.04	.025	.159	0.10 *	0.04	.026	.163
D3: FPB → CAW	0.01	0.03	−.057	.078	0.02	0.03	−.053	.080

*Notes*. * *p* < .05. *SE* = standard error, LLCI = the lower limit of 95% confidence interval, ULCI = the upper limit of 95% confidence interval, FPB = father progressive belief, FI = father involvement, FCCLS = father–child closeness, FCCOT = father–child conflict, CSC = child social competence, CAA = child anger–aggression, CAW = child anxiety–withdrawal.

## Data Availability

Data will be made available upon reasonable request.
